# Implications of a new clinical classification of acute myocardial infarction

**DOI:** 10.1093/ehjacc/zuaf002

**Published:** 2025-01-18

**Authors:** Jasper Boeddinghaus, Anda Bularga, Caelan Taggart, Ryan Wereski, Michael McDermott, Alexander J F Thurston, Amy V Ferry, Michelle C Williams, Andrew H Baker, Marc R Dweck, David E Newby, Andrew R Chapman, Bertil Lindahl, Nicholas L Mills, DEMAND-MI Investigators, Anda Bularga, John Hung, Marwa Daghem, Stacey Schulberg, Caelan Taggart, Ryan Wereski, Trisha Singh, Mohammed N Meah, Takeshi Fujisawa, Amy V Ferry, Justin Chiong, William S Jenkins, Fiona E Strachan, Scott Semple, Edwin J R van Beek, Michelle C Williams, Damini Dey, Chris Tuck, Andrew H Baker, David E Newby, Marc R Dweck, Nicholas L Mills, Andrew R Chapman

**Affiliations:** BHF/University Centre for Cardiovascular Science, University of Edinburgh, Edinburgh EH16 4SA, UK; Cardiovascular Research Institute Basel (CRIB) and Department of Cardiology, University Hospital Basel, University of Basel, Basel CH-4056, Switzerland; BHF/University Centre for Cardiovascular Science, University of Edinburgh, Edinburgh EH16 4SA, UK; BHF/University Centre for Cardiovascular Science, University of Edinburgh, Edinburgh EH16 4SA, UK; BHF/University Centre for Cardiovascular Science, University of Edinburgh, Edinburgh EH16 4SA, UK; BHF/University Centre for Cardiovascular Science, University of Edinburgh, Edinburgh EH16 4SA, UK; BHF/University Centre for Cardiovascular Science, University of Edinburgh, Edinburgh EH16 4SA, UK; BHF/University Centre for Cardiovascular Science, University of Edinburgh, Edinburgh EH16 4SA, UK; BHF/University Centre for Cardiovascular Science, University of Edinburgh, Edinburgh EH16 4SA, UK; BHF/University Centre for Cardiovascular Science, University of Edinburgh, Edinburgh EH16 4SA, UK; BHF/University Centre for Cardiovascular Science, University of Edinburgh, Edinburgh EH16 4SA, UK; BHF/University Centre for Cardiovascular Science, University of Edinburgh, Edinburgh EH16 4SA, UK; BHF/University Centre for Cardiovascular Science, University of Edinburgh, Edinburgh EH16 4SA, UK; Department of Medical Sciences, Uppsala University, Uppsala 751 85, Sweden; BHF/University Centre for Cardiovascular Science, University of Edinburgh, Edinburgh EH16 4SA, UK; Usher Institute, University of Edinburgh, Edinburgh EH16 4UX, UK

**Keywords:** Myocardial infarction, Cardiac troponin, Imaging, Coronary artery disease

## Abstract

**Aims:**

The diagnostic criteria for Type 2 myocardial infarction identify a heterogeneous group of patients with variable outcomes and no clear treatment implications. We aimed to determine the implications of a new clinical classification for myocardial infarction with more objective diagnostic criteria using cardiac imaging.

**Methods and results:**

In a prospective cohort study, patients with Type 2 myocardial infarction underwent coronary angiography and cardiac magnetic resonance imaging or echocardiography. The new classification was applied to identify (i) spontaneous myocardial infarction due to acute coronary pathology, (ii) secondary myocardial infarction precipitated by acute illness in the presence of obstructive coronary artery disease, a new regional wall motion abnormality, or infarct-pattern scarring, and (iii) no myocardial infarction in the absence of obstructive disease or new myocardial abnormality. In 100 patients (65 years, 43% women) with Type 2 myocardial infarction, the new classification identified 25 and 31 patients with spontaneous and secondary myocardial infarction, respectively, and 44 without myocardial infarction. Compared with patients without myocardial infarction, those with secondary myocardial infarction were older, had more risk factors, and had higher troponin concentrations (*P* < 0.05 for all). During a median follow-up of 4.4 years, death, myocardial infarction, or heart failure hospitalization was more common in secondary myocardial infarction compared with those without myocardial infarction [55% (17/31) vs. 16% (7/44), *P* < 0.001].

**Conclusion:**

A new clinical classification of myocardial infarction informed by cardiac imaging would reduce the diagnosis of myocardial infarction in acute illness and identify those patients at highest risk who are most likely to benefit from treatment.

**Clinical trial registration:**

https://clinicaltrials.gov/ct2/show/NCT03338504.

## Introduction

The Universal Definition of Myocardial Infarction recognizes different subtypes of myocardial infarction,^1^ where Type 1 occurs in patients with atherothrombosis and Type 2 in patients where there is evidence of myocardial oxygen supply-demand imbalance without atherothrombosis.^1–3^ The definition of Type 2 myocardial infarction includes a broad range of coronary and non-coronary mechanisms, and consequently, outcomes are highly variable.^4–7^ Whilst this approach to classifying patients with myocardial infarction according to aetiology has been an important conceptual advance, the decision to group coronary and non-coronary mechanisms into a single classification of Type 2 myocardial infarction was based on consensus rather than prospective studies and the implications of this diagnosis for patients remain unclear.^1,8^ Evidence-based management of Type 1 myocardial infarction is widely adopted, but major uncertainty remains regarding the diagnosis, management, and outcomes of patients with Type 2 myocardial infarction.^8–17^ As a result, the diagnosis has not been applied consistently in clinical practice.^18^

An alternative clinical classification for myocardial infarction has been proposed to address the problems associated with Type 2 myocardial infarction.^19^ This new classification recognizes that myocardial infarction can arise in only three settings: (i) spontaneously due to acute coronary pathology, (ii) secondary to another acute condition, or (iii) as a procedural complication following coronary intervention or cardiac surgery. The majority of patients with spontaneous myocardial infarction have atherothrombosis and are managed consistently unless angiography identifies an alternative coronary mechanism, such as spontaneous coronary artery dissection. In patients with myocardial ischaemia or injury during acute illness, the new classification encourages the use of cardiac imaging and proposes more objective diagnostic criteria for secondary myocardial infarction. Where acute illness unmasks obstructive coronary artery disease or causes a pattern of functional impairment or myocardial scarring consistent with myocardial infarction, the diagnosis of secondary myocardial infarction is appropriate.^19^

In a prospective cohort study of patients meeting the diagnostic criteria for Type 2 myocardial infarction,^9^ our aim was to evaluate how a new clinical classification with more objective diagnostic criteria for secondary myocardial infarction using cardiac imaging would impact the diagnosis and identification of those at risk of adverse outcomes.

## Methods

### Study design

Secondary analysis of a prospective observational cohort study (clinicaltrials.gov NCT03338504).^9^ The study was approved by the South-east Scotland Regional Ethics Committee and conducted in accordance with the Declaration of Helsinki with written informed consent.

### Study population

The study protocol was published previously.^9^ In brief, consecutive patients presenting to the Royal Infirmary of Edinburgh, Scotland, in whom the attending clinician requested a cardiac troponin measurement, were screened using a tool embedded in the electronic patient record.^20,21^ The study team continuously reviewed the electronic patient record in all patients with an elevated cardiac troponin concentration to identify those who met the diagnostic criteria for Type 2 myocardial infarction.^1^ Patients were eligible if they had evidence of a rise and/or fall in cardiac troponin with at least one value above the sex-specific 99th centile, and symptoms or signs on the 12-lead electrocardiogram of myocardial ischaemia in whom there was objective evidence of myocardial oxygen supply or demand imbalance (see Supplementary material). We did not enrol patients with suspected Type 1 myocardial infarction, women who were pregnant or breastfeeding, those with renal impairment or severe hepatic impairment, or those with advanced frailty and inability to self-transfer.^22^

### Study procedures and imaging

Details of the study procedures and imaging protocols were reported previously^9^ and are available in the data supplement (see Supplementary material). In brief, coronary angiography was performed by invasive catheterization or computed tomography (CT) depending on co-morbidities and patient preference. Coronary CT angiography was performed using a 128-multidetector CT scanner (Siemens Biograph, Siemens Healthcare, Erlangen, Germany). Cardiac magnetic resonance imaging was performed using a 3T scanner (MAGNETOM Verio, Siemens AG, Healthcare Sector, Erlangen, Germany), and transthoracic echocardiography was performed according to national guidelines.^23^

### Independent diagnostic adjudication and reclassification

Following cardiac imaging, all patients with a clinical diagnosis of Type 2 myocardial infarction prior to enrolment underwent independent review by an adjudication panel. All clinical data and study imaging were used to adjudicate the diagnosis by consensus according to the Fourth Universal Definition of Myocardial Infarction.^1^ For this study, patients were also classified according to a new clinical classification of myocardial infarction.^19^ Patients found to have atherothrombosis or another acute coronary pathology (spontaneous dissection, embolism, vasospasm, in-stent restenosis, late stent thrombosis, or late graft failure) were reclassified as spontaneous myocardial infarction, applying the same clinical criteria for the diagnosis as in the Fourth Universal Definition of Myocardial Infarction.^1^ Patients where an alternative acute illness resulting in supply-demand imbalance unmasked obstructive coronary artery disease, or resulted in a pattern of functional myocardial impairment (regional wall motion abnormality in a coronary distribution) or scarring [infarct pattern late gadolinium enhancement (LGE)] consistent with myocardial infarction were reclassified as secondary myocardial infarction. Obstructive coronary artery disease was defined as a stenosis >50% in the left main stem or >70% in a major epicardial vessel. Where patients had known left ventricular impairment, previous imaging was reviewed to identify new abnormalities. Patients who did not have obstructive coronary artery disease, or new functional consequences of myocardial injury in the context of acute illness were reclassified as not having a myocardial infarction (see Supplementary material).

### Study outcomes

The diagnostic outcome was the proportion of patients with a clinical diagnosis of Type 2 myocardial infarction reclassified as having spontaneous, secondary, or no myocardial infarction by the new clinical classification. The prognostic outcome was a composite of all-cause death, any recurrent myocardial infarction, or heart failure hospitalization during follow-up to 30 October 2023. All subsequent myocardial infarction events were adjudicated.

### Statistical analysis

We compared characteristics in all patients with a clinical diagnosis of Type 2 myocardial infarction and stratified according to the groups proposed by the new clinical classification of myocardial infarction. The frequency of the primary prognostic outcome was assessed using the Kaplan–Meier estimate, with the log-rank test for comparisons. Group-wise comparisons were performed using Fisher’s exact, *χ*^2^, Kruskal–Wallis or one-way analysis of variance tests as appropriate. All analyses were performed in R (version 3.5.1).

## Results

Between January 2018 and October 2020, 108 patients who met the criteria for a diagnosis of Type 2 myocardial infarction were consented. Of these, eight patients did not attend for investigations or withdrew consent. The final study population comprised 100 patients [median age 65 (55–74) years, 43% women] with a clinical diagnosis of Type 2 myocardial infarction (see Supplementary material online, *Figure S1*).

### Characteristics of patients reclassified with and without myocardial infarction

**Figure 1:**
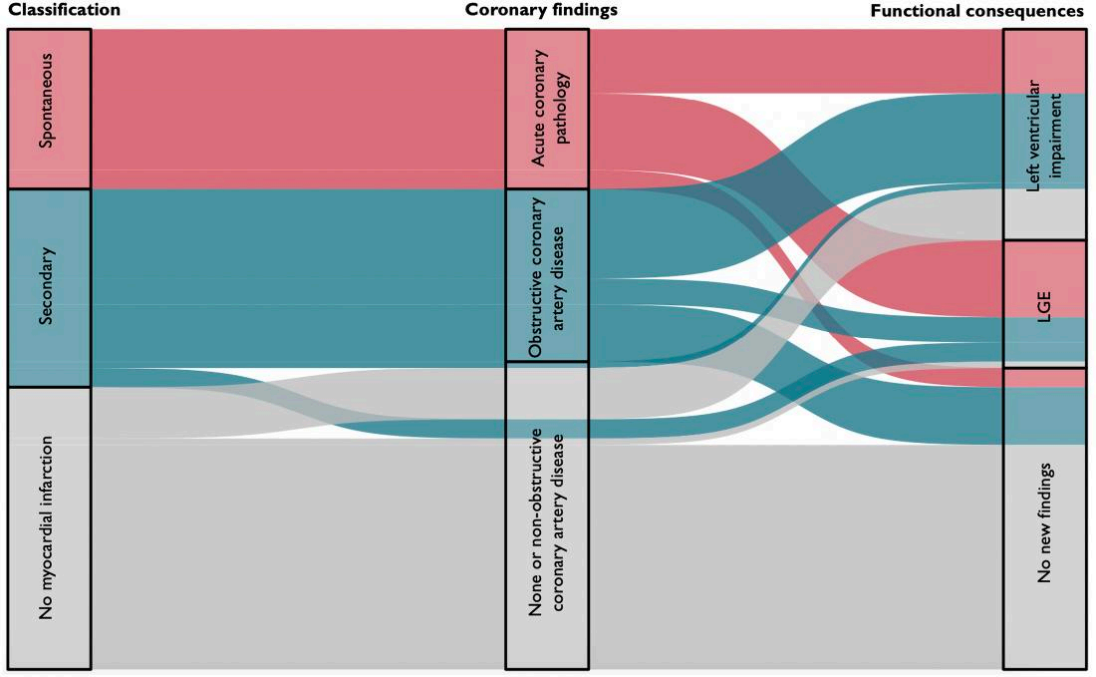
Alluvial plot illustrating the reclassification of patients with a diagnosis of Type 2 myocardial infarction into spontaneous and secondary myocardial infarction or no myocardial infarction. Patients with spontaneous myocardial infarction had evidence of acute coronary pathology (acute atherothrombosis, coronary embolism, spontaneous coronary dissection, and coronary vasospasm). Patients with secondary myocardial infarction had either evidence of obstructive coronary artery disease or functional consequences of myocardial injury with new left ventricular systolic impairment or evidence of infarct pattern late gadolinium enhancement on cardiac magnetic resonance imaging with or without regional wall motion abnormality. Two patients classified as no myocardial infarction had evidence of previous infarction with late gadolinium enhancement.

In 100 patients with a clinical diagnosis of Type 2 myocardial infarction, application of the new clinical classification would reclassify 25 patients as spontaneous myocardial infarction and 44 as no myocardial infarction, with 31 patients meeting the diagnostic criteria for secondary myocardial infarction (*Figure 1*; Supplementary material online, *Figure S1*). Compared with patients without myocardial infarction, those with secondary myocardial infarction were older, had more cardiovascular risk factors, had higher cardiac troponin concentrations, and more often had a history of myocardial infarction (*Table 1*). In contrast, those with spontaneous myocardial infarction were younger and less likely to have risk factors.

**Table 1:** Baseline characteristics in patients with a clinical diagnosis of Type 2 myocardial infarction reclassified as having spontaneous myocardial infarction, secondary myocardial infarction, or no myocardial infarction

	Type 2 myocardial infarction	Clinical classification of myocardial infarction			*P*-values^a^		
		Spontaneous	Secondary	No infarction	Secondary vs. spontaneous	Secondary vs. no infarction	Spontaneous vs. no infarction
Number of participants	100	25	31	44	—	—	—
Age (years)	65 (55–74)	55 (49–63)	73 (67–80)	64 (55–73)	<0.001	0.001	0.018
Female	43 (43)	12 (48)	9 (29)	22 (50)	0.200	0.100	>0.900
Current or previous cigarette smoker	41 (41)	10 (40)	14 (45)	17 (39)	0.800	0.600	>0.900
Primary presenting symptom							
Chest pain	70 (70)	23 (92)	18 (58)	29 (66)	0.015	0.200	0.084
Dyspnoea	9 (9)	0 (0)	6 (19)	3 (7)			
Palpitations	9 (9)	0 (0)	2 (7)	7 (16)			
Syncope	10 (10)	2 (8)	5 (16)	3 (7)			
Other	2 (2)	0 (0)	0 (0)	2 (5)			
Medical history							
Diabetes mellitus	11 (11)	3 (12)	7 (23)	1 (2)	0.500	0.007	0.130
Hypercholesterolaemia	19 (19)	6 (24)	7 (23)	6 (14)	>0.900	0.400	0.300
Hypertension	42 (42)	8 (32)	17 (55)	17 (39)	0.110	0.200	0.600
Myocardial infarction	15 (15)	5 (20)	8 (26)	2 (5)	0.800	0.013	0.090
Cerebrovascular disease	4 (4)	1 (4)	3 (10)	0 (0)	0.600	0.067	0.400
Atrial fibrillation	16 (16)	2 (8)	7 (23)	7 (16)	0.200	0.600	0.500
Heart failure	3 (3)	0 (0)	3 (10)	0 (0)	0.200	0.067	—
Chronic obstructive pulmonary disease	11 (11)	2 (8)	6 (19)	3 (7)	0.300	0.150	>0.900
Other chronic respiratory illness	11 (11)	4 (16)	3 (10)	4 (9)	0.700	>0.900	0.400
Malignancy	10 (10)	1 (4)	5 (16)	4 (9)	0.200	0.500	0.600
Prior revascularization							
PCI	11 (11)	2 (8)	7 (23)	2 (5)	0.200	0.028	0.600
CABG surgery	8 (8)	0 (0)	7 (23)	1 (2)	0.013	0.007	>0.900
Medications at presentation							
Aspirin	27 (27)	7 (28)	14 (45)	6 (14)	0.300	0.003	0.200
P2Y_12_ inhibitor	13 (13)	5 (20)	5 (16)	3 (7)	0.700	0.300	0.130
Lipid-lowering therapy	37 (37)	9 (36)	18 (58)	10 (23)	0.120	0.003	0.300
Beta-blocker	31 (31)	7 (28)	14 (45)	10 (23)	0.300	0.048	0.800
ACE inhibitor or ARB	40 (40)c	10 (40)	14 (45)	16 (36)	0.800	0.500	0.800
Nitrates	25 (25)	8 (32)	13 (42)	4 (9)	0.600	0.002	0.022
Oral anticoagulant	16 (16)	2 (8)	7 (23)	7 (16)	0.200	0.600	0.500
Admission clinical parameters							
Heart rate, b.p.m.	99 (77–130)	84 (59–102)	98 (81–129)	110 (85–160)	0.059	0.200	0.005
Systolic blood pressure, mmHg	126 (110–154)	128 (124–156)	130 (109–143)	124 (109–160)	0.400	0.800	0.300
Oxygen saturation, %	97 (96–99)	98 (96–99)	96 (92–98)	97 (96–98)	0.080	0.200	0.800
Respiratory rate, breaths/min	18 (16–20)	17 (16–18)	18 (18–23)	18 (16–20)	0.010	0.110	0.300
Temperature, °C	36.5 (36.3–37.0)	36.8 (36.3–37.2)	36.5 (36.3–37.0)	36.5 (36.1–37.0)	0.400	>0.900	0.400
Admission electrocardiogram							
Rhythm							
Sinus	63 (63)	20 (80)	20 (65)	23 (52)	0.600	0.700	0.200
Second or third degree AV block	1 (1)	0 (0)	0 (0)	1 (2)			
Atrial fibrillation/flutter	24 (24)	4 (16)	7 (23)	13 (30)			
Supraventricular tachycardia	5 (5)	0 (0)	1 (3)	4 (9)			
Ventricular arrhythmia	7 (7)	1 (4)	3 (10)	3 (7)			
Myocardial ischaemia	78 (78)	25 (100)	24 (77)	29 (66)	0.013	0.300	<0.001
ST-segment elevation	18 (18)	13 (52)	3 (10)	2 (5)	<0.001	0.600	<0.001
ST-segment depression	31 (31)	6 (24)	11 (35)	14 (32)	0.400	0.800	0.600
T-wave inversion	44 (44)	13 (52)	15 (48)	16 (36)	>0.900	0.300	0.200
Bundle branch block	10 (10)	1 (4)	3 (10)	6 (14)	0.600	0.700	0.400
Haematology and clinical chemistry							
Haemoglobin, g/L	138 (128–151)	142 (131–152)	131 (96–138)	142 (130–152)	0.004	0.002	>0.900
White cell count, ×10^9^/L	9.4 (7.5–11.7)	9.8 (6.3–11.3)	10.6 (8.0–14.4)	9.1 (7.1–10.6)	0.130	0.063	>0.900
Platelet count, ×10^9^/L	237 (206–288)	236 (216–275)	266 (208–314)	234 (200–276)	0.300	0.130	0.600
Urea, mmol/L	5.8 (4.4–7.1)	4.3 (3.1–5.6)	7.0 (5.1–9.1)	6.2 (4.9–6.8)	<0.001	0.200	<0.001
Creatinine, mmol/L	80 (68–104)	71 (63–84)	107 (72–126)	76 (69–89)	0.004	0.016	0.300
Admission hs-cTnI, ng/L	200 (62–810)	347 (93–1319)	181 (83–1288)	142 (51–564)	0.500	0.400	0.100
Maximum hs-cTnI, ng/L	1292 (277–4043)	6451 (707–18 136)	1522 (914–3780)	578 (96–2151)	0.200	0.008	<0.001

The values are given as median (interquartile range) and number (%).

ACE, angiotensin-converting enzyme; ARB, angiotensin receptor blocker; AV, atrioventricular; CABG, coronary artery bypass graft; hs-cTnI, high-sensitivity cardiac troponin I; PCI, percutaneous coronary intervention.

^a^Between-group comparisons of patients according to reclassification group are Fisher’s exact test or Wilcoxon rank-sum test.

Compared with patients without myocardial infarction, those with secondary myocardial infarction had similar clinical presentations with 66 and 58% reporting chest pain, and 66 and 77% having evidence of myocardial ischaemia on the electrocardiogram, respectively, suggesting that these features are not discriminatory (*Table 1*). In contrast, the majority of patients reclassified as having spontaneous myocardial infarction reported chest pain (92%), and all had evidence of myocardial ischaemia on the electrocardiogram. The maximal cardiac troponin concentration was higher in spontaneous and secondary myocardial infarction compared with those without myocardial infarction [6451 (707–18,136) and 1522 (914–3780) vs. 578 (96–2151) ng/L, *P* < 0.001 for both].

### Cardiac imaging in patients reclassified with and without myocardial infarction

In patients reclassified as having spontaneous myocardial infarction, 96% underwent invasive coronary angiography, whereas CT coronary angiography was performed in 42 and 55% of those reclassified as secondary myocardial infarction or no myocardial infarction, respectively (*Table 2*). The majority of patients underwent cardiac magnetic resonance imaging (78%) with similar proportions across the groups (spontaneous 80%, secondary 81%, and no myocardial infarction 75%), but where this was not feasible or tolerated, echocardiography was performed.

**Table 2:** Coronary and cardiac imaging findings in patients with a clinical diagnosis of Type 2 myocardial infarction reclassified as having spontaneous myocardial infarction, secondary myocardial infarction, or no myocardial infarction

	Type 2 myocardial infarction	Clinical classification of myocardial infarction		
		Spontaneous	Secondary	No infarction
Number of participants	100	25	31	44
Coronary imaging				
CT coronary angiography	38 (38)	1 (4)	13 (42)	24 (55)
Invasive coronary angiography	62 (62)	24 (96)	18 (58)	20 (45)
Atherosclerotic coronary artery disease				
Coronary artery disease	68 (68)	15 (60)	30 (97)	23 (52)
Stenosis severity				
Non-obstructive	37 (37)	11 (44)	3 (1)	23 (52)
Mild (<50%)	29 (29)	10 (40)	3 (1)	16 (36)
Moderate (50–70%)	8 (8)	1 (4)	0 (0)	7 (16)
Obstructive	32 (32)	5 (20)	27 (87)	0 (0)
One vessel	14 (14)	4 (16)	10 (32)	0 (0)
Two vessels	12 (12)	1 (4)	11 (35)	0 (0)
Three vessels	6 (6)	0 (0)	6 (19)	0 (0)
Left main stem	3 (3)	0 (0)	3 (10)	0 (0)
Proximal LAD	18 (18)	2 (8)	16 (52)	0 (0)
Structural imaging				
Cardiac magnetic resonance imaging	78 (78)	20 (80)	25 (81)	33 (75)
Transthoracic echocardiogram	22 (22)	5 (20)	6 (19)	11 (25)
Evidence of myocardial infarction				
Infarct-pattern LGE^a^	36 (36)	19 (95)	15 (60)	2 (6)
Subendocardial^a^	24 (31)	9 (45)	14 (56)	1 (3)
Transmural^a^	12 (16)	10 (50)	1 (4)	1 (3)
Regional wall motion abnormality	30 (30)	15 (60)	11 (35)	4 (9)
Structural heart disease				
Dilated cardiomyopathy	2 (2)	0 (0)	0 (0)	2 (5)
Hypertrophic cardiomyopathy	2 (2)	0 (0)	1 (3)	1 (2)
Ischaemic cardiomyopathy	10 (10)	2 (8)	7 (23)	1 (2)
Hypertensive heart disease	8 (8)	0 (0)	1 (3)	7 (16)
Myocarditis	1 (1)	0 (0)	0 (0)	1 (2)
Non-ischaemic cardiomyopathy	5 (5)	0 (0)	1 (3)	4 (9)
Takotsubo cardiomyopathy	1 (1)	0 (0)	0 (0)	1 (2)
Valvular heart disease	13 (13)	3 (13)	6 (19)	4 (9)
LV function assessment				
Normal LV function (EF ≥55%)	67 (67)	15 (60)	16 (52)	36 (82)
Mild LV impairment (EF 45–54%)	17 (17)	9 (36)	5 (16)	3 (7)
Moderate LV impairment (EF 35–44%)	10 (10)	0 (0)	7 (23)	3 (7)
Severe LV impairment (EF <35%)	6 (6)	1 (4)	3 (10)	2 (5)

The values are given as number (%).

CAD, coronary artery disease; CT, computed tomography; LAD, left anterior descending; LGE, late gadolinium enhancement; LV, left ventricle.

^a^Proportion of those who underwent cardiac magnetic resonance imaging.

**Figure 2:**
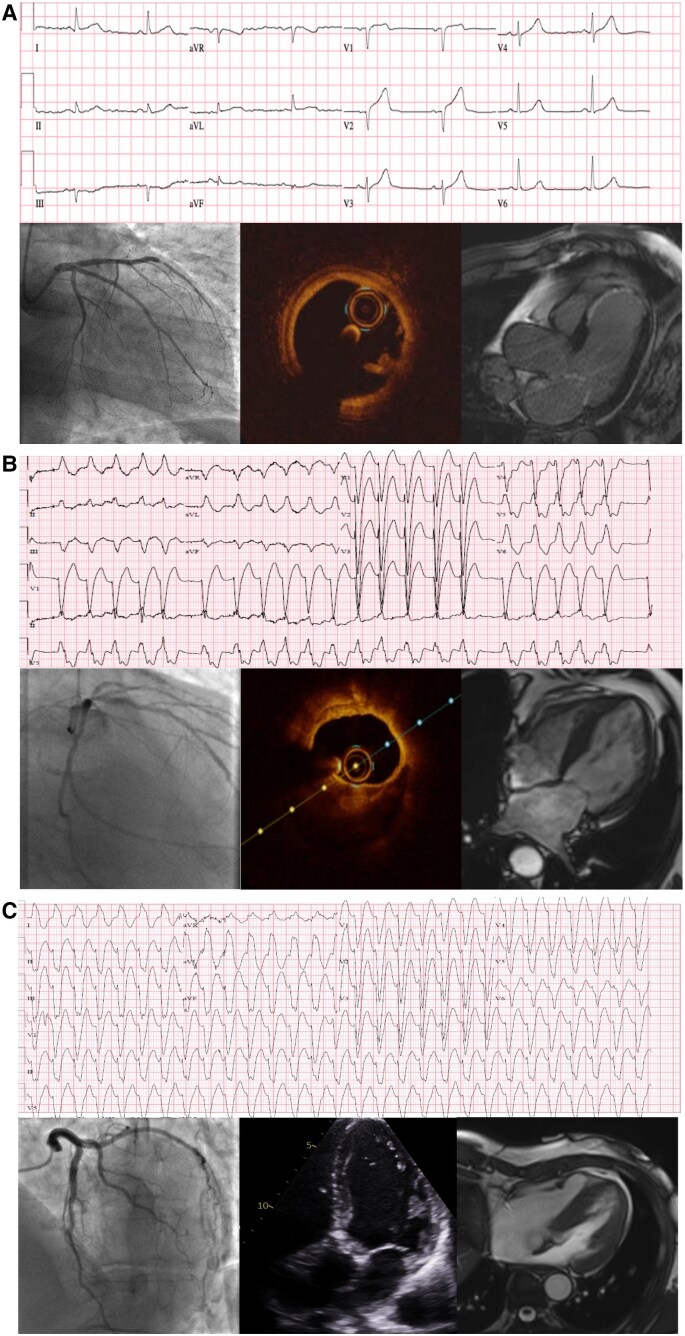
Case of spontaneous myocardial infarction (*A*), secondary myocardial infarction (*B*), and no myocardial infarction (*C*). (*A*) A 65-year-old patient with a recent diagnosis of atrial fibrillation and prior history of hypertension, Type 2 diabetes mellitus, and chronic obstructive pulmonary disease presented with chest pain at rest. They were found to be in sinus rhythm with ST-segment elevation across the anterior leads. Coronary angiography demonstrated abrupt occlusion of the left anterior descending artery, and thrombectomy was performed to restore coronary perfusion. Optical coherence tomography imaging demonstrated minimal atherosclerosis with no evidence of plaque rupture, and the patient was anticoagulated with apixaban. The peak cardiac troponin I concentration was 32 201 ng/L. Cardiac magnetic resonance imaging revealed near transmural late gadolinium enhancement in the mid-to-apical antero-septum with evidence of left ventricular systolic impairment (ejection fraction 48%). The final diagnosis was spontaneous myocardial infarction secondary to coronary embolism. (*B*) An 82-year-old patient with a history of angina and peripheral vascular disease presented with sudden onset palpitation and chest pain. The electrocardiogram demonstrated rapid atrial fibrillation with left bundle brunch block. Peak cardiac troponin I concentration was 3500 ng/L. Coronary angiography demonstrated diffuse coronary artery disease with a severe ostial lesion of the left anterior descending coronary artery. Optical coherence tomography revealed no evidence of plaque rupture or thrombosis. Pressure wire assessment showed marked gradient across the lesion (fractional flow reserve 0.99–0.56). Cardiac magnetic resonance imaging revealed severe left ventricular systolic impairment (ejection fraction 16%). The scan was stopped early on patient request and late gadolinium enhancement sequences were not obtained. The final diagnosis was secondary myocardial infarction due to atrial fibrillation with obstructive coronary artery disease and significant left ventricular impairment. (*C*) A 72-year-old patient with hypertension and hypercholesterolaemia presented with a 2 day history of chest tightness, left arm pain, and palpitations. The electrocardiogram demonstrated ventricular tachycardia, and the patient required cardioversion on arrival. The peak cardiac troponin I concentration was 1637 ng/L. Coronary angiography demonstrated normal coronary arteries with evidence of marked calcification of the aortic valve and aortic root dilatation. The patient underwent an echocardiogram which showed evidence of severe bicuspid aortic stenosis. Cardiac magnetic resonance imaging identified normal left ventricular systolic function (ejection fraction 67%) with no late gadolinium enhancement. Myocardial infarction was excluded. The final diagnosis was ventricular tachycardia secondary to severe aortic stenosis.

In patients reclassified as spontaneous myocardial infarction due to the identification of acute coronary pathology (*Figure 1*), this was due to acute atherothrombosis (*n* = 5), coronary embolism (*n* = 8), spontaneous coronary dissection (*n* = 7), and coronary vasospasm (*n* = 5). In those reclassified as secondary myocardial infarction, this was based on the presence of obstructive coronary artery disease in 87% (27/31). By definition, patients without myocardial infarction cannot have obstructive coronary artery disease, but around half had evidence of non-obstructive coronary artery disease. Examples are given to illustrate how the new clinical classification was applied to reclassify patients with spontaneous (*Figure 2A*), secondary (*Figure 2B*), or no myocardial infarction (*Figure 2C*).

The majority of patients reclassified without myocardial infarction had normal left ventricular function without an infarct pattern of LGE. In patients reclassified with spontaneous myocardial infarction, 40% (10/25) had left ventricular systolic impairment, with the majority having mild dysfunction. Left ventricular impairment was observed in 48% (15/31) of patients reclassified as secondary myocardial infarction, but in contrast, impairment was mild, moderate, or severe in 16, 23, and 10%, respectively. In patients who underwent cardiac magnetic resonance imaging, there was evidence of infarct-pattern LGE in 95% (19/20) and 60% (15/25) of patients with spontaneous and secondary myocardial infarction, respectively, which was more likely to be transmural in the former (*Table 2*). In patients reclassified as secondary myocardial infarction, just 45 and 58% were established on aspirin or a lipid-lowering therapy, 45% were on a beta-blocker, and 45% were on an angiotensin-converting enzyme inhibitor (*Table 1*).

### Outcomes according to the new classification of myocardial infarction

**Figure 3:**
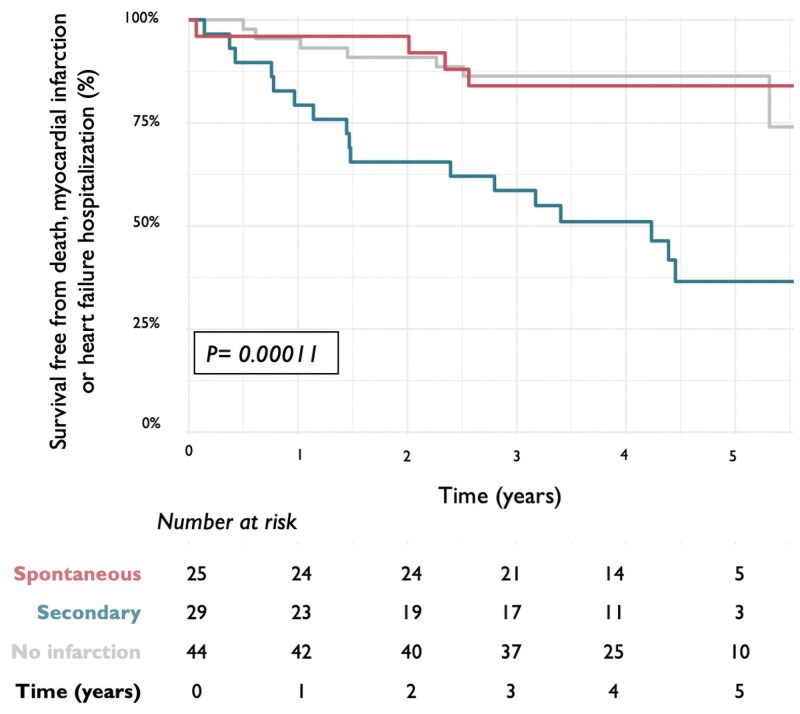
Survival free from death, myocardial infarction, or heart failure hospitalization in patients with spontaneous and secondary myocardial infarction and those without myocardial infarction. Kaplan–Meier curves illustrating survival free from all-cause death, myocardial infarction, or hospitalization with heart failure in patients with a clinical diagnosis of Type 2 myocardial infarction reclassified following cardiac imaging as having spontaneous, secondary, or no myocardial infarction according to the new clinical classification.

The median time to follow-up was 4.4 (interquartile range 3.9–5.1) years and was complete for 98 (98%) patients. Patients with secondary myocardial infarction had a higher rate of death, subsequent myocardial infarction, or hospitalization with heart failure compared with those without myocardial infarction [55% (17/31) vs. 16% (7/44), Fisher’s exact test *P* < 0.001; *Figure 3*, Supplementary material online, *Table S2* and *Figure S2*].

## Discussion

In this prospective study of patients with a clinical diagnosis of Type 2 myocardial infarction undergoing systematic cardiac imaging, we determined the potential implications of a new clinical classification of myocardial infarction on diagnosis, management, and outcomes.

We report several findings that will inform discussion around the merits of this classification. First, when applying the new clinical classification in patients with Type 2 myocardial infarction, one in four were reclassified as spontaneous myocardial infarction and in almost half, myocardial infarction was excluded as injury was not due to coronary artery disease or associated with functional consequences. As such, just one in three patients currently classified as Type 2 myocardial infarction according to the Universal Definition would receive a diagnosis of secondary myocardial infarction. Second, patients that were reclassified as secondary myocardial infarction were older, more likely to have cardiovascular risk factors, and had higher cardiac troponin concentrations. Third, half of the patients reclassified as secondary myocardial infarction were on no preventative therapy prior to undergoing cardiac imaging, suggesting that a classification that promotes the use of imaging could identify opportunities to target evidence-based therapies for coronary artery disease and left ventricular impairment. Fourth, patients with secondary myocardial infarction were at increased risk of death, subsequent myocardial infarction and heart failure hospitalization compared with those without myocardial infarction. Together, these findings suggest the application of a new clinical classification of myocardial infarction would identify patients at increased risk of adverse outcomes who may benefit from secondary prevention.

The Universal Definition of Myocardial Infarction has been an important achievement in modern medicine.^1^ The task force has achieved a global consensus with support from many of the major cardiac societies and World Health Foundation, promoted standardization of the diagnostic criteria, supported sex-specific cardiac troponin thresholds to reduce inequalities in care, and encouraged clinicians to consider the mechanism of myocardial infarction and to tailor the treatment accordingly. These achievements have undoubtedly improved the quality of care and outcomes for many patients.^24^ However, there remain major challenges in adopting some of the current recommendations in practice.

The diagnosis of Type 2 myocardial infarction was introduced in 2007 in recognition that cardiac troponin concentrations were elevated in many patients without atherothrombosis.^25^ The diagnostic criteria prioritize sensitivity and apply the same cardiac troponin threshold as for Type 1 myocardial infarction. The diagnosis of Type 2 myocardial infarction can therefore be made in any patient with myocardial oxygen supply-demand imbalance, a rise and/or fall in cardiac troponin with one value above the 99th centile, in whom there are symptoms or signs of myocardial ischaemia.^1^ As a result, the diagnosis of Type 2 myocardial infarction can be made without cardiac imaging or objective evidence of myocardial ischaemia.^26^ Anecdotally cardiologists often use this term to describe patients who in their judgement do not require further investigation or care by a cardiologist.^27^ The other challenge is that the current definition of Type 2 myocardial infarction encompasses a very broad range of patients with different mechanisms of supply-demand imbalance who need different treatments and have divergent clinical outcomes,^4,5,28^ from acute coronary mechanisms such as coronary artery dissection, embolism, and vasospasm, to multiple cardiac or systemic illnesses that cause tachycardia, hypotension, hypoxia, or anaemia. The diagnosis can even be made in patients without coronary artery disease, despite this being the pathological basis of myocardial infarction since the 19th century.^29^ As a result, the implications of a diagnosis of Type 2 myocardial infarction are uncertain for both patients and clinicians, and this has led to a limited adoption of the classification in practice and a paucity of research into care pathways that could potentially improve outcomes. A new classification could overcome this and improve patient understanding of how their cardiac diagnosis relates to their acute illness and why this has implications for their care.

In practice, patients with a spontaneous presentation are assumed to have atherothrombosis unless coronary angiography identifies an alternative acute coronary pathology. Whether the presentation is due to atherothrombosis or an alternative acute coronary pathology is impossible to determine without performing coronary angiography. The diagnostic criteria need to be highly sensitive to ensure early diagnosis and treatment for all those with acute coronary pathologies. Separating spontaneous myocardial infarction due to an acute coronary pathology from myocardial infarction arising in the context of supply-demand imbalance in acute illness enables the use of sensitive diagnostic criteria for the former and more specific diagnostic criteria for the later. Adoption should encourage the use of diagnostic coronary angiography in patients with spontaneous presentations to clarify the underlying mechanism and treatment tailored accordingly.

The new clinical classification should also promote the use of cardiac imaging in those where the presentation is secondary to an acute illness to determine whether the criteria for myocardial infarction are met. In practice, echocardiography would be the first-line investigation in the majority of patients, with magnetic resonance imaging considered in those where diagnostic uncertainty remains. Unless there are ongoing symptoms or signs of myocardial ischaemia, or there is uncertainty as to whether the presentation was due to an acute coronary pathology, then coronary imaging could be deferred until the acute illness has resolved. In this setting, it is likely that non-invasive imaging with CT coronary angiography will play an increasing role. Coronary imaging may not be necessary where the clinician considers the patient to have a low probability of underlying coronary artery disease. It is important to highlight that patients in whom secondary myocardial infarction has been excluded are also likely to benefit from the proposed classification, as cardiac imaging may identify unrecognized non-ischaemic cardiomyopathies, myocarditis, or valvular disease.^9^

Whilst one in three patients with a clinical diagnosis of Type 2 myocardial infarction in our cohort met the criteria for secondary myocardial infarction, in practice, the proportion is likely to be smaller. Furthermore, the proportion of patients with a clinical diagnosis of Type 2 myocardial infarction, in whom cardiac imaging would result in reclassification as spontaneous myocardial infarction due to an acute coronary pathology is likely to be substantially lower than one in four in practice. Whilst we systematically screened all patients with elevated cardiac troponin concentrations identifying >700 consecutive patients who met the diagnostic criteria for Type 2 myocardial infarction, only 100 patients met our inclusion and exclusion criteria, and therefore, our findings are not representative of the broader patient population. The main reason for exclusion was frailty where cardiac imaging may not be in the patients’ interest. Referral bias may have enriched the study population. We anticipate that in a less selected population the proportion in whom myocardial infarction is excluded is likely to be greater and the proportion with spontaneous myocardial infarction due to mechanisms other than atherothrombosis is likely to be smaller.

There are some additional limitations that merit consideration. First, we enrolled patients with a clinical diagnosis of Type 2 myocardial infarction. Given we observed no differences in the proportion of patients with chest pain or myocardial ischaemia on the electrocardiogram in those with and without secondary myocardial infarction, the proposed clinical classification of myocardial infarction would benefit from further evaluation in prospective studies enrolling a broader population of patients with acute myocardial injury. Second, our sample size is small and does not allow for subgroup analyses. Although differences in outcomes between those with and without secondary myocardial infarction were observed, larger prospective studies are needed to confirm this observation. Third, not all patients underwent cardiac magnetic resonance imaging due to contraindications, patient choice, and public health restrictions during the pandemic. Whilst transthoracic echocardiography was performed in the remainder and is likely to be the main imaging method used in clinical practice, we may have underestimated the prevalence of new regional wall motion abnormalities and secondary myocardial infarction in our study population. Finally, further prospective studies are needed to determine the costs of applying a new clinical classification in practice that encourages the use of cardiac imaging.

## Conclusions

A new clinical classification of myocardial infarction informed by cardiac imaging would reduce the diagnosis of myocardial infarction in acute illness and identify those patients at highest risk who are most likely to benefit from treatment.

## Determining the mechanism of myocardial injury and role of coronary disease in Type 2 myocardial infarction (DEMAND-MI) investigators to be listed as contributors

Anda Bularga, John Hung, Marwa Daghem, Stacey Schulberg, Caelan Taggart, Ryan Wereski, Trisha Singh, Mohammed N. Meah, Takeshi Fujisawa, Amy V. Ferry, Justin Chiong, William S. Jenkins, Fiona E. Strachan (BHF Centre for Cardiovascular Science, University of Edinburgh, UK); Scott Semple, Edwin J. R. van Beek, Michelle C. Williams (Edinburgh Imaging, University of Edinburgh, UK); Damini Dey (Biomedical Imaging Research Institute, Cedars-Sinai Medical Center, Los Angeles, USA); Chris Tuck, Andrew H. Baker, David E. Newby, Marc R. Dweck, Nicholas L. Mills, MD, Andrew R. Chapman (BHF Centre for Cardiovascular Science, University of Edinburgh, UK).

## Supplementary Material

zuaf002_Supplementary_Data

## Data Availability

The data that support the findings of this study are available from the corresponding author upon reasonable request.
