# Supplementary material for: Implications of a new clinical classification of acute myocardial infarction
Source: Eur Heart J Acute Cardiovasc Care. 2025 Jan 18;14(3):131–41. doi: 10.1093/ehjacc/zuaf002 (PMC11929527; doi:10.1093/ehjacc/zuaf002)
Supplement: zuaf002_Supplementary_Data [file zuaf002_supplementary_data.docx]

##### SUPPLEMENTARY MATERIAL

Implications of a new clinical classification

of acute myocardial infarction

Jasper Boeddinghaus, MD,^1,2*^ Anda Bularga, MD,^1*^ Caelan Taggart, MD,^1^

Ryan Wereski, MD,^1^ Michael McDermott, MD,^1^ Alexander JF Thurston, MD,^1^

Amy V Ferry, PhD, Michelle C Williams, MD PhD,^1^ Andrew H Baker, PhD,^1^

Marc R Dweck, MD PhD,^1^ David E Newby, MD PhD,^1^Andrew R Chapman, MD PhD,^1#^ Bertil Lindahl, MD,^3#^ Nicholas L Mills, MD PhD,^1,4#^

on behalf of the DEMAND-MI Investigators^†^

^1^ BHF/University Centre for Cardiovascular Science, University of Edinburgh, Edinburgh, UK

^2^ Cardiovascular Research Institute Basel (CRIB) and Department of Cardiology, University Hospital Basel, University of Basel, Switzerland

^3^ Department of Medical Sciences, Uppsala University, Uppsala, Sweden

^4^ Usher Institute, University of Edinburgh, Edinburgh, UK

^*^Contributed equally

^#^Contributed equally

^†^Investigators listed at the end of the article

**Short title:** A new classification of myocardial infarction

**Key words:** myocardial infarction, cardiac troponin, imaging, coronary artery disease
**X:** @HighSTEACS @J_Boeddinghaus @andabularga @chapdoc1

**Corresponding author:**

Professor Nicholas L Mills

BHF/University Centre for Cardiovascular Science

The University of Edinburgh

Royal Infirmary of Edinburgh

Edinburgh EH16 4SA

United Kingdom

Telephone: 0044 131 242 6515

E-mail: [nick.mills@ed.ac.uk](mailto:nick.mills@ed.ac.uk)

**Supplementary Methods**

**Study population**

Consecutive patients were enrolled with acute myocardial injury (defined as a rise and or fall in cardiac troponin concentration on serial testing, with at least one value >99^th^ centile) in whom symptoms or signs of myocardial ischemia on the electrocardiogram were observed and the mechanism of injury was myocardial oxygen supply and demand imbalance.^1^ Signs of myocardial ischemia were defined as new ST-segment elevation or depression or T-wave inversion changes in-keeping with acute ischemia using pre-defined criteria.^1^ Consecutive patients were identified through screening cardiac troponin measurements using the electronic patient record and laboratory databases at the recruiting site. All screened patients were recorded in a screening log. Patients who meet both the inclusion and exclusion criteria, were approached and those who provided consent comprised the study population and were allocated a unique study number.

**Inclusion criteria:**

- Unscheduled hospital admission with acute myocardial injury defined as a rise and or fall in cardiac troponin concentration with at least one value >99^th^ centile
- Myocardial oxygen supply and demand imbalance with symptoms or signs of myocardial ischemia

**Exclusion criteria:**

- Unable or unwilling to give informed consent
- Women who are pregnant or breastfeeding will not be enrolled into the trial.
- Probable type 1 myocardial infarction
- Renal impairment (estimated glomerular filtration rate ≤30 ml/min/1.73m^2^)
- Severe hepatic impairment
- Frailty with inability to self-transfer determined using Katz Index

**Imaging Protocols**

**Coronary imaging**

Invasive coronary angiography was performed via the radial or femoral approach. In patients with one or more stenoses in a major epicardial vessel, a coronary pressure wire (PressureWire™ Aeris™, St. Jude Medical, St. Paul, Minnesota) assessment was undertaken to determine distal coronary pressure and the fractional flow reserve (FFR) calculated at maximal adenosine-induced (intravenous 140 μg/kg/min) hyperaemia.^2^ Optical coherence tomography (OCT) was performed where possible with pullback at 20 mm/s to identify features consistent with vulnerable plaque or recent plaque rupture.^3^

Coronary computed tomography angiography was performed using a 128 multidetector CT scanner (Siemens Biograph, Siemens Healthcare, Erlangen, Germany). Patients with a heart rate exceeding 65 beats/min were administered oral or intravenous beta-blockade and all received sublingual glyceryl trinitrate (300μg) immediately prior to electrocardiogram-gated coronary computed tomography angiography during breath-hold. A bolus of 80-100 mL of contrast (400 mg/mL; Iomeron, Bracco, Milan, Italy) was injected intravenously at 5 mL/s.

**Cardiac imaging**

Cardiac magnetic resonance imaging (MRI) was performed in all patients without contraindication using a 3T scanner (MAGNETOM Verio, Siemens AG, Healthcare Sector, Erlangen, Germany). The MRI scan consisted of localisers, axial and coronal HASTE images, and standard breath-held and ECG-gated cine sequences. These were acquired with standard steady-state free precession sequences in long- and short-axis orientations as described previously.^4^ The late gadolinium enhancement and T2-weighted (MyoMaps) imaging were used to identify regions of new or old myocardial infarction as well as other patterns of injury. T2 measurements were taken from septal segments and from the site of late gadolinium enhancement where this was present. Reference T2 values were obtained from a cohort of ten healthy volunteers who underwent cardiac MRI on the same 3T scanner (mean T2 value 38.7±3 ms).^5^ Where feasible and coronary anatomy was known with no contraindications present, patients underwent stress perfusion imaging following intravenous administration of a 0.2 mmol/kg Gadolinium contrast bolus and 0.4mg (5ml) of Regadenoson (Rapiscan™) 8-15 minutes post first contrast administration. Late gadolinium enhancement images were acquired in 2 chamber and 4 chamber short and long-axis views and in two phase encoding directions. T2 mapping (MyoMaps) was acquired in the 2 chamber and 4 chamber long-axis orientation. The late gadolinium enhancement and T2-weighted imaging techniques were used to identify regions of new or old myocardial infarction as well as other patterns of injury.

There were three major components to the cardiac magnetic resonance imaging protocol:

| **Major components** | **Structural imaging** | **T1-weighted coronary imaging and T2 mapping** | **Late gadolinium enhancement** |
| --- | --- | --- | --- |
| Pulse sequence | Cine MRI | Pre contrast imaging | Fast IR-prepared gradient echo post contrast |
| Typical images | 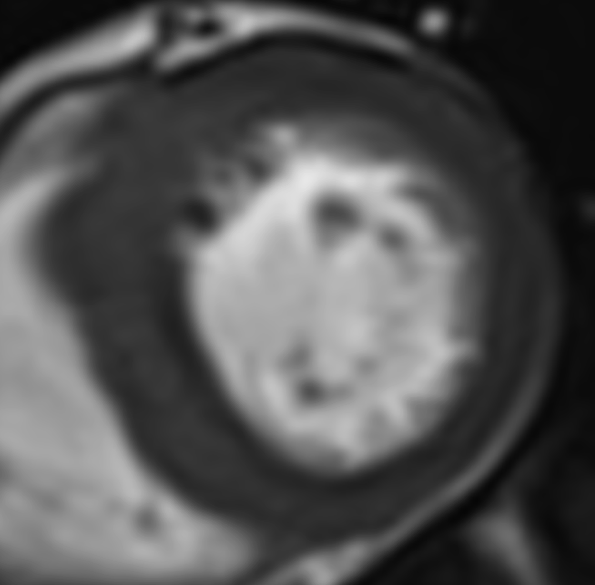 | 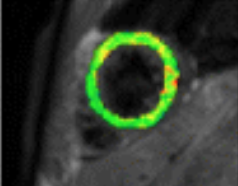 | 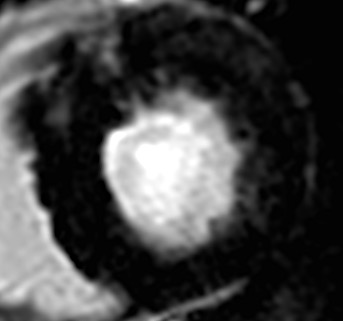 |
| Rationale | Quantify LV size, mass and systolic function | To assess for coronary thrombus and myocardial oedema (above) | Identify the presence of mid-wall late gadolinium enhancement as a marker of LV decompensation |

The imaging protocol consisted of:

1. Localiser sequences
2. Left ventricular imaging (4 chambers, 2 chambers and 3 chambers long axis cines)
3. Interleaved angiogram and T1-weighted coronary imaging (CATCH)
4. T2 myocardial mapping (2, 3 and 4 chamber long axis images)
5. Stress perfusion imaging (with *Regadenoson*)
6. Gadolinium contrast administration
7. LV (left ventricular) short axis cine stack
8. Late gadolinium enhancement imaging

**Table of cardiac MRI sequences:**

|  | Number of acquisitions | Estimated time | Time post-contrast |
| --- | --- | --- | --- |
| Position subject within scanner, ensure high quality ECG signal and IV cannula patency |  | 5 mins |  |
| Sequence |  |  |  |
| Localiser | 1 |  |  |
| Site specific method of localisers to derive appropriate cardiac planes | 1-5 | 1-3 mins |  |
| 4 chamber (horizontal long axis) cine | 1 | 2 min |  |
| 2 chamber (vertical long axis) cine | 1 | 2 min |  |
| 3 chamber cine | 1 | 2 min |  |
| Interleaved angiogram and T1-weighted coronary imaging (CATCH sequence) | 1 | 12 mins |  |
| T2 myocardial mapping  (2, 3 and 4 chamber long axis images) | 3 | 3 mins |  |
| Inject regadenoson |  |  |  |
| Inject contrast (0.15mmol/kg) |  |  |  |
| Stress perfusion imaging  (short axis images, basal mid and apex) |  | 3 mins | 1-3 |
| LV short axis stack cine – 8mm slice thickness, no gap | 10 | 4 mins | 3-7 |
| TI scout – to determine optimal TI | 1 | 1 min | 7-8 |
| GRE-T1 LGE LV short axis stack – 8mm slice thickness, no gap | 14-15 | 7 mins | 8-15 |
| Phase swap GRE-T1 LGE LV short axis stack* | 14-15 | 7 mins | 15-22 |
| Long axis GRE-T1 LGE images | 3 | 3 mins | 22-25 |
| Total scan time |  |  |  |
| Standard protocol |  | 55 mins |  |

**Image Analysis**

**Coronary imaging**

Coronary computed tomography angiography (CCTA) images were reviewed on a dedicated post processing workstation (Vitrea Advanced, v6.9.68.1, Vital Images, US) by experienced observers (MCW, EJRB), who performed a per-segment analysis using a 15-segment model to assess coronary artery stenoses, with complex cases classified by consensus. Reconstructions of contrast-enhanced images were performed on the best phase in mid-diastole or end-systole based on established techniques.^6^ Luminal cross-sectional area stenoses were classified as normal (<10%), mild non-obstructive (10%-49%), moderate non-obstructive (50%-70%), or obstructive (>70% in ≥1 major epicardial artery or >50% in the left main stem).^6,7^

**Cardiac imaging**

Cardiac magnetic resonance studies were analysed offline using Circle CVI (Circle Cardiovascular Imaging, CVI42 v5.3.6, Calgary Canada). T2 maps, and cine-derived volumetric and functional sequences was analysed by experienced observers (MRD, WJ, ARC, JH, MD, TS, AB). Endocardial and epicardial borders were manually defined on all the conventional short-axis images for volumetric and wall motion measurements and were then copied to corresponding LGE sequences for analysis with minimal manual adjustments.^4^ Regions of interest (ROIs) were determined using the standard 16-segment cardiac model with septal native T2 values. For patients with previous myocardial infarction or a new diagnosis of infarction, the region of interest (ROIs) was defined in the remote myocardium. We determined left ventricular ejection fraction using standard volumetric analysis of a short axis stack sequence. A blinded analysis of cardiac MRI was undertaken by an independent expert (WJ), to validate the assessment of late gadolinium enhancement pattern. The blinded analysis involved assessment of late gadolinium enhancement presence and pattern (ischaemic or non-ischaemic [mid-wall or epicardial]).

Echocardiography was undertaken in line with guidance from the British Society of Echocardiography.^8^ Standard image acquisition including basal dimensions, an assessment of ventricular function with estimated left ventricular ejection fraction using Simpson’s biplane, and assessment of valvular dysfunction using standard doppler imaging was obtained in line with requirements for a standard examination.

**Supplementary Tables**

**Supplementary Table 1. The Strengthening the Reporting of Observational Studies in Epidemiology (STROBE) checklist**

|  | Item No | Recommendation | Page  No |
| --- | --- | --- | --- |
| **Title and abstract** | 1 | (*a*) Indicate the study’s design with a commonly used term in the title or the abstract | p1 |
|  |  | (*b*) Provide in the abstract an informative and balanced summary of what was done and what was found | p2 |
| Introduction | | | |
| Background/rationale | 2 | Explain the scientific background and rationale for the investigation being reported | p7 |
| Objectives | 3 | State specific objectives, including any prespecified hypotheses | p6-7 |
| Methods | | | |
| Study design | 4 | Present key elements of study design early in the paper | p5-7 |
| Setting | 5 | Describe the setting, locations, and relevant dates, including periods of recruitment, exposure, follow-up, and data collection | p6-7 |
| Participants | 6 | (*a*) *Cohort study*—Give the eligibility criteria, and the sources and methods of selection of participants. Describe methods of follow-up | P6-7 |
| Variables | 7 | Clearly define all outcomes, exposures, predictors, potential confounders, and effect modifiers. Give diagnostic criteria, if applicable | p7 |
| Data sources/ measurement | 8* | For each variable of interest, give sources of data and details of methods of assessment (measurement). Describe comparability of assessment methods if there is more than one group | P6-7 |
| Bias | 9 | Describe any efforts to address potential sources of bias | p6-7 |
| Study size | 10 | Explain how the study size was arrived at | p6-7 |
| Quantitative variables | 11 | Explain how quantitative variables were handled in the analyses. If applicable, describe which groupings were chosen and why | p6-7 |
| Statistical methods | 12 | (*a*) Describe all statistical methods, including those used to control for confounding | p7 |
|  |  | (*b*) Describe any methods used to examine subgroups and interactions | p7 |
|  |  | (*d*) *Cohort study*—If applicable, explain how loss to follow-up was addressed | p7 |

| Results | | | |
| --- | --- | --- | --- |
| Participants | 13* | (a) Report numbers of individuals at each stage of study—eg numbers potentially eligible, examined for eligibility, confirmed eligible, included in the study, completing follow-up, and analysed | p8 |
|  |  | (b) Give reasons for non-participation at each stage | p8 |
|  |  | (c) Consider use of a flow diagram | Supp F1 |
| Descriptive data | 14* | (a) Give characteristics of study participants (eg demographic, clinical, social) and information on exposures and potential confounders | p8, Table 1 |
|  |  | (b) Indicate number of participants with missing data for each variable of interest | NA |
|  |  | (c) *Cohort study*—Summarise follow-up time (eg, average and total amount) | p10 |
| Outcome data | 15* | *Cohort study*—Report numbers of outcome events or summary measures over time | Supp Table 2 |
| Main results | 16 | (*a*) Give unadjusted estimates and, if applicable, confounder-adjusted estimates and their precision (eg, 95% confidence interval). Make clear which confounders were adjusted for and why they were included | NA |
|  |  | (*b*) Report category boundaries when continuous variables were categorized | p8-10, Table 1 |
|  |  | (*c*) If relevant, consider translating estimates of relative risk into absolute risk for a meaningful time period | NA |
| Discussion | | | |
| Key results | 18 | Summarise key results with reference to study objectives | p11 |
| Limitations | 19 | Discuss limitations of the study, taking into account sources of potential bias or imprecision. Discuss both direction and magnitude of any potential bias | p13-14 |
| Interpretation | 20 | Give a cautious overall interpretation of results considering objectives, limitations, multiplicity of analyses, results from similar studies, and other relevant evidence | p12-15 |
| Generalisability | 21 | Discuss the generalisability (external validity) of the study results | p12-15 |
| Other information | | | |
| Funding | 22 | Give the source of funding and the role of the funders for the present study and, if applicable, for the original study on which the present article is based | p17 |

.

**Supplementary Table 2. Distribution of outcome events during follow up according to the new classification of myocardial infarction**

|  | Type 2 myocardial infarction | Clinical classification of myocardial infarction | | | |
| --- | --- | --- | --- | --- | --- |
|  |  | **Spontaneous** | **Secondary** | **No infarction** | **Secondary vs**  **No infarction** |
| Number of participants | 100 | 25 | 31 | 44 | - |
| Primary outcome^§^ | 28 (28) | 4 (16) | 17 (55) | 7 (16) | <0.001 |
| All-cause death* | 18 (18) | 3 (12) | 14 (45) | 1 (2) | <0.001 |
| Cardiovascular death | 3 (3) | 2 (8) | 4 (13) | 0 (0) | 0.067 |
| Non-cardiovascular death | 12 (12) | 1 (4) | 10 (30) | 1 (2) | <0.001 |
| Myocardial infarction | 14 (14) | 2 (8) | 7 (23) | 5 (11) | 0.065 |
| Universal Definition of Myocardial Infarction | | | | | |
| Type 1 myocardial infarction | 5 (5) | 1 (4) | 4 (13) | 0 (0) | - |
| Type 2 myocardial infarction | 9 (9) | 1 (4) | 3 (10) | 5 (11) | - |
| Classification of myocardial infarction | | | | | |
| Spontaneous | 5 (5) | 1 (4) | 4 (13) | 0 (0) | - |
| Secondary | 4 (4) | 1 (4) | 3 (10) | 0 (0) | - |
| No infarction | 5 (5) | 0 (0) | 0 (0) | 5 (11) | - |
| Revascularization | 2 (2) | 0 (0) | 2 (7) | 0 (0) | 0.026 |
| Heart failure hospitalization | 8 (8) | 1 (4) | 6 (19) | 1 (2) | 0.003 |
| Number (%). Overall, complete follow up was available in 98 patients.  ^§^Primary outcome is a composite of all-cause death, any recurrent myocardial infarction, or heart failure hospitalization.  *The cause of death was unknown in three patients and in these cases the death was assumed to be cardiovascular.  Myocardial infarction events were adjudicated according to the Fourth Universal Definition of Myocardial Infarction and the new clinical classification.  **Between-group comparisons of patients according to reclassification group are Fisher's exact test.* | | | | | |

**Supplementary Figures**

**Supplementary Figure 1. Patient flow**

**
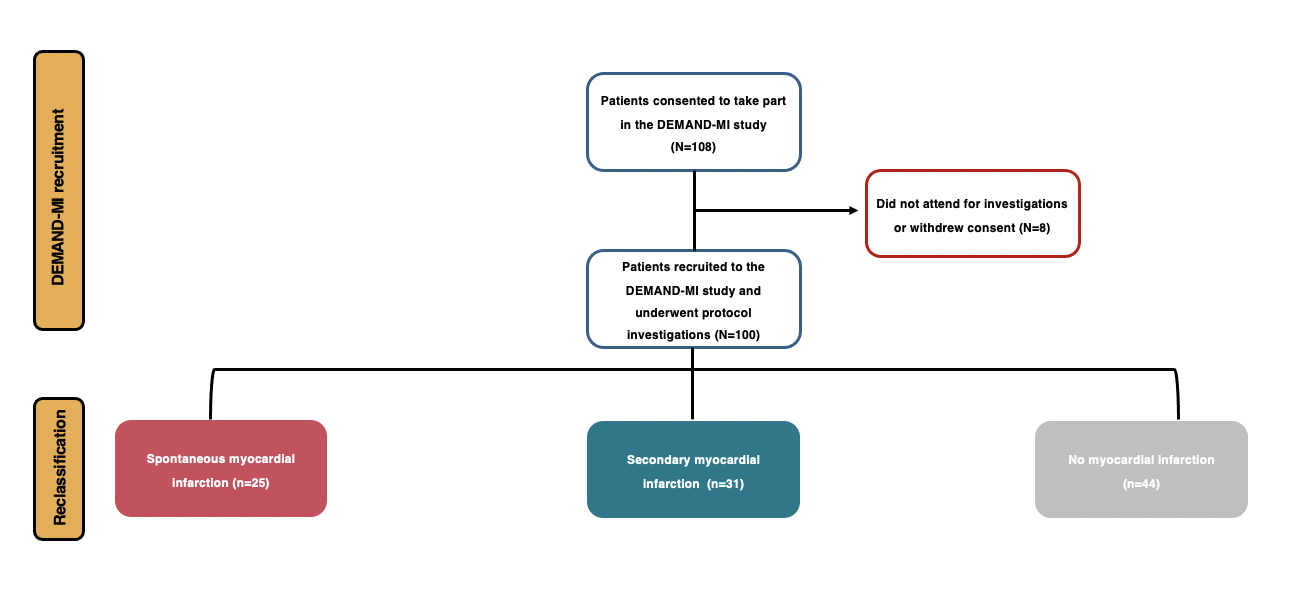
**

**
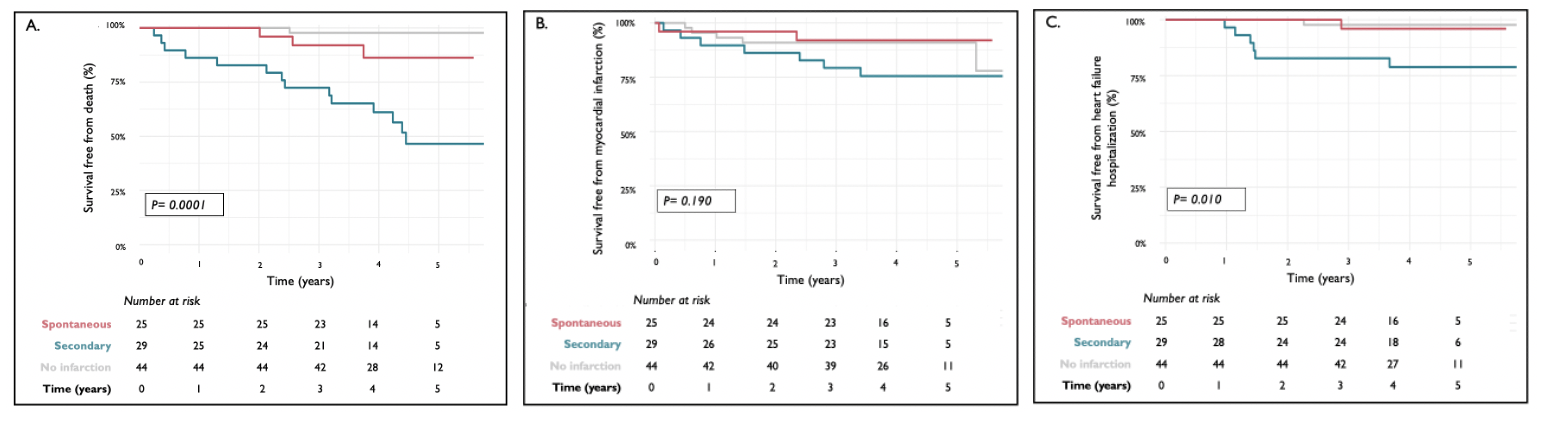
Supplementary Figure 2. Survival free from death (A), myocardial infarction (B), and heart failure hospitalization (C) in patients with spontaneous and secondary myocardial infarction and those without myocardial infarction**

**References**

1. Thygesen K, Alpert JS, Jaffe AS, et al. Fourth Universal Definition of Myocardial Infarction (2018). *Circulation* 2018; 138: e618–e651.

2. Achenbach S, Rudolph T, Rieber J, et al. Performing and Interpreting Fractional Flow Reserve Measurements in Clinical Practice: An Expert Consensus Document. *Interventional Cardiology Review* 2017; 12: 97.

3. Gudmundsdottir I, Adamson P, Gray C, et al. Optical coherence tomography versus intravascular ultrasound to evaluate stent implantation in patients with calcific coronary artery disease. *Open Heart* 2015; 2: e000225.

4. Kramer CM, Barkhausen J, Flamm SD, et al. Standardized cardiovascular magnetic resonance (CMR) protocols 2013 update. *Journal of Cardiovascular Magnetic Resonance* 2013; 15: 1–10.

5. Singh T, Kite TA, Joshi SS, et al. MRI and CT coronary angiography in survivors of COVID-19. *Heart (British Cardiac Society)* 2022; 108: 46–53.

6. Abbara S, Blanke P, Maroules CD, et al. SCCT guidelines for the performance and acquisition of coronary computed tomographic angiography: A report of the society of Cardiovascular Computed Tomography Guidelines Committee: Endorsed by the North American Society for Cardiovascular Imaging (NASCI). *Journal of Cardiovascular Computed Tomography* 2016; 10: 435–449.
